# Active roles of dysfunctional vascular endothelium in fibrosis and cancer

**DOI:** 10.1186/s12929-019-0580-3

**Published:** 2019-10-28

**Authors:** Tien Hsu, Hieu-Huy Nguyen-Tran, Maria Trojanowska

**Affiliations:** 1 Department of Biomedical Sciences and Engineering, National Central University, 300 Jhongda Rd, Taoyuan City, Taiwan Republic of China; 2 Center for Chronic Disease Research, National Central University, 300 Jhongda Rd, Taoyuan City, Taiwan Republic of China; 3 Arthritis Center, Boston University School of Medicine, 75 E. Newton St. Evans Building, Boston, MA 02118 USA

**Keywords:** Inflammation, Endothelial cells, Fibrosis, Cancer vasculature

## Abstract

Chronic inflammation is the underlying pathological condition that results in fibrotic diseases. More recently, many forms of cancer have also been linked to chronic tissue inflammation. While stromal immune cells and myofibroblasts have been recognized as major contributors of cytokines and growth factors that foster the formation of fibrotic tissue, the endothelium has traditionally been regarded as a passive player in the pathogenic process, or even as a barrier since it provides a physical divide between the circulating immune cells and the inflamed tissues. Recent findings, however, have indicated that endothelial cells in fact play a crucial role in the inflammatory response. Endothelial cells can be activated by cytokine signaling and express inflammatory markers, which can sustain or exacerbate the inflammatory process. For example, the activated endothelium can recruit and activate leukocytes, thus perpetuating tissue inflammation, while sustained stimulation of endothelial cells may lead to endothelial-to-mesenchymal transition that contributes to fibrosis. Since chronic inflammation has now been recognized as a significant contributing factor to tumorigenesis, it has also emerged that activation of endothelium also occurs in the tumor microenvironment. This review summarizes recent findings characterizing the molecular and cellular changes in the vascular endothelium that contribute to tissue fibrosis, and potentially to cancer formation.

## Background

Endothelial cells (ECs) are specialized cells that line both large and small blood vessels throughout the body. They play an important role in the coagulation cascade, inflammation, maintenance of blood pressure and angiogenesis. The most recognizable function of the endothelium is to maintain a barrier between the bloodstream and tissue, while allowing limited exchange of cellular and molecular materials. Such function is vital to its role of delivering oxygen and nutrients to, and transporting metabolic wastes from internal organs. Upon breach of the barrier function, the endothelium promotes thrombosis and fibrinolysis; that is, the formation of blood clots. Thus, formation of new blood vessels (neoangiogenesis) is critical during embryonic organ development and in tissue repair and wound healing [1]. Neoangiogenesis is a well-coordinated complex process resulting in formation of functional blood vessels. Uncontrolled excessive angiogenesis contributes to the development of inflammatory diseases including rheumatoid arthritis (RA), psoriasis, atopic dermatitis, and inflammatory bowel disease (IBD), as well as tumor formation [2]. In contrast, diseases associated with tissue fibrosis, often considered the result of chronic inflammation, are accompanied by loss of vasculature. It is therefore important to consider whether vasculature is a passive responder or plays an important effector role in inflammatory and fibrotic diseases.

In fact, accumulated evidence has implicated the dysfunctional or activated endothelium in many of the immune-related diseases. Dysfunctional endothelium is broadly defined as endothelial cells exhibiting functional changes that lead to a shift from homeostasis towards proinflammatory response, reduced vasodilation, and proliferative and prothrombotic properties. In the following sections, we will discuss the pathogenic features in these diseases and the involvement of vascular endothelium. It should be noted that lymphatic endothelium likely is also an important player in inflammation, but here we will focus on vascular endothelium, the function of which is better elucidated. We will then discuss the endothelial cell response during chronic inflammation at the cellular and molecular levels, and discuss whether these events are also relevant in cancer formation, which is now considered an immune disorder.

### Wound repair

The normal body response to tissue injury, which is best studied in the skin, involves rapid influx of inflammatory cells, proliferation and migration of epithelial cells, expansion of fibroblasts and endothelial cell populations, formation of granulation tissue followed by the deposition of extracellular matrix (ECM), and in the final phase, matrix remodeling and scar formation [3]. Initial injury to the tissue leads to immediate activation of the clotting cascade, which, through the assembly of a fibrin clot, assures hemostasis and provides the basic matrix architecture to initiate the invasion and recruitment of inflammatory and other cells. In this process, formation of new blood vessels is indispensable for proper repair and involves both sprouting of capillaries from existing vessels and mobilization of bone-marrow endothelial progenitor cells. In the well-controlled wound healing, inflammation resolves quickly and the cells that contribute to tissue repair, but no longer needed, undergo apoptosis. Many pathological conditions such as inflammatory and fibrotic diseases are frequently compared to perpetual wound healing with the former characterized by the non-resolving early inflammatory phase and the latter by the excessive reparative phase. Analysis of chronic wound tissue suggested a persistent competition between inflammatory and anti-inflammatory signals leading to a continuously unstable microenvironment unfavorable for proper wound healing [4]. It has been shown that increased infiltration of proinflammatory leukocytes such as neutrophils and macrophages contribute to delayed healing in chronic ulcers [5, 6] by secreting interleukin (IL)-1β and tumor necrosis factor–α (TNF-α) [7].

The need for neoangiogenesis in wound healing is supported by the observations that pathological conditions associated with insufficient angiogenesis such as diabetes mellitus or systemic sclerosis (scleroderma or SSc) are defective in wound healing with less tissue granulation and ineffective wound maturation [8]. In normal wound healing, after an injury, levels of proangiogenic factors such as VEGF-A, TGF-β, and FGF2 increase, coinciding with a maximum capillary content, and then subside to nearly undetectable levels during the final phase of wound repair. Thus, neoangiogenesis in wound healing is a dynamic process, and is at least in part downstream of tissue inflammation, since macrophages have been shown to be a major angiogenic inducer [9, 10]. At latter stage of wound healing, the neovessels are subjected to vascular pruning until most of the newly formed vessels regress to a vessel density comparable to that of normal, uninjured skin. The process includes the selective apoptosis of many of the newly formed capillaries, followed by maturation of the remaining ones, including coverage by pericytes [11]. During this complex process, it is apparent that endothelial cells can respond to many signaling factors and undergo physiological transformation, including proliferation, cell shape change, increased motility, altered adhesion capability, interaction with leukocytes, cell death, among others. However, not all these changes can be categorized as branching and sprouting that are usually described as angiogenic response. The underlying mechanisms that regulate these cellular events, and their relevance to tissue inflammation will be discussed in the latter sections.

### Inflammatory disorders

Chronic inflammatory disorders such as RA, psoriasis, IBD, and several others are typically associated with excessive angiogenesis [12–15]. Although of different etiology, these disorders are all characterized by persistent injury leading to continuous early phase of the tissue repair process and uncontrolled inflammation. The cause of persistent injury can be the result of persistent infection, continuous physical damage, or autoimmunity. Below, RA is used as a representative inflammatory disorder to describe in more details the pathogenic processes characterizing this group of diseases.

In RA, a prototypic immune mediated inflammatory disorder, synovium is the primary injury site [16]. Healthy synovium consists of the lining layer made of fibroblast-like synoviocytes with interspersed macrophage-like synoviocytes and a network of vascular capillaries embedded in the connective tissue located underneath. Osteoclasts, the primary bone resorption cells, are located in the synovium at sites adjacent to bone [17]. Osteoclasts together with osteoblasts are responsible for bone remodeling during physiological bone repair processes.

The triggers of the disease are currently not well defined, but immunological abnormalities, e.g., presence of the anticitrullinated protein antibodies, precedes the development of RA [16]. Early stages of the disease are characterized by increased proliferation of the synovial lining cells, increased angiogenesis, and infiltration of the immune cells. The key cytokines driving the inflammatory process in RA include TNF-α, IL-1β, IL-6, IL-17, and GM-CSF [18]. Among these cytokines, TNF-α plays a central role in propagating joint inflammation and bone destruction. TNF-α stimulates osteoclastogenesis and promotes survival of osteoclasts while inhibiting differentiation of osteoblasts. Activated macrophages and fibroblasts are the primary producers of TNF-α, but other cell types, including T and B cells, osteoblasts and osteoclasts constitute the additional source [19]. Activated fibroblasts also contribute to cartilage destruction by producing excessive amounts of matrix-degrading enzymes such as MMP1, −3 and −13, as well as cathepsin K and L [19]. Besides immune cells, different subgroups of fibroblasts also play distinct immune and bone effector functions that contribute to different disease characteristics in RA [20, 21]. The roles of fibroblasts in RA have been reviewed extensively elsewhere [22, 23] and will not be covered here.

Macrophages constitute the dominant immune cell population in the inflamed joint and the major producer of inflammatory cytokines, including TNF-α, IL-6, and IL-1β. In RA, macrophages undergo a metabolic switch from oxidative phosphorylation to glycolysis resulting in activation of the proinflammatory transcription factors such as HIF-1α, STAT3 and NF-kB, and increased production of inflammatory cytokines [24–26]. An important bi-directional cross-talk between synovial fibroblasts and macrophages contributing to the disease pathogenesis has been recently described [27]. The authors used single-cell RNA sequencing to characterize macrophages in RA synovium. The predominant macrophages subset termed “Cluster 1 HBEGF+” exhibits characteristics distinct from the classical M1/M2 phenotypes with the expression of NR4A3, PLAUR, CXCL2, HBEGF, and epiregulin (EREG). The unique phenotype of these macrophages was dependent on the factors secreted by RA fibroblasts, primarily prostaglandins and TNF-α. In turn, the HBEGF+ macrophages, which produced EGF receptor ligands, HB-EGF and epiregulin, induced invasive phenotype in fibroblasts.

Dysfunctional endothelial cells have been observed in RA patients more than a decade ago and have been linked to the increased risk of atherosclerosis in these patients [28–30]. Recently, Totoson et al. have demonstrated that endothelial activation and restoration of homeostatic endothelium closely follow the progression and recovery of arthritis in an experimental murine mode; the endothelial activation is mediated by the COX-2 pathway and induced by inflammatory cytokines such as TNF-α, IL-1β and MIP-1α [31].

Thus, during inflammatory response in RA, inflammatory cytokines such as TNFα, IL-1β, and IL-6 appear to play critical roles. Interestingly, receptors or co-receptors for these cytokines are highly expressed in ECs [32–34], indicating that inflammatory response can also be induced in ECs.

### Fibrotic disorders

Pathological fibrosis, defined as the excessive deposition of collagen and other extracellular matrix proteins, is commonly characterized by the maladaptive repair process in response to tissue injury. Although the molecular and cellular mechanism of fibrosis is not yet fully elucidated, and may vary depending on the trigger and the anatomy of the affected organ, immune cells, endothelial or epithelial cells, and fibroblasts/myofibroblasts are the principal contributors to this process in different organs. For the purpose of this review we will discuss fibrotic disorders that are triggered by the EC injury such as scleroderma.

In scleroderma (systemic sclerosis, SSc), microvasculature is the primary injury site. Diverse triggers implicated in inducing EC damage include infection, immune-mediated cytotoxicity, anti-endothelial autoantibodies (AECAs), and ischemia-reperfusion injury [35]. Progressive structural damage of capillaries in the absence of compensatory angiogenesis and vasculogenesis results in systemic rarefaction of the microvasculature and tissue hypoxia. Although the factors contributing to vessel rarefaction in scleroderma are not fully understood, one of the proposed causes is an imbalance between the angiogenic and angiostatic factors [36]. For example, although the levels of the key angiogenic growth factor VEGF-A are increased in SSc, the balance between proangiogenic VEGF_165_ and its anti-angiogenic splice variant VEGF_165b_ is tilted towards VEGF_165b_ [37]. Furthermore, CXCL4, one of the most potent anti-angiogenic chemokines, is highly elevated in scleroderma [38]. Anti-angiogenic effects of CXCL4 are mediated through downregulation of transcription factor Fli1. Fli1 and its close homolog ERG are considered central regulators of angiogenesis and expression of both factors is decreased in SSc vasculature [39, 40]. Other mediators elevated in SSc and implicated in downregulation of Fli1 include IFN-α and TGF-β [41, 42]. Toll-like receptor ligands induce their anti-angiogenic response via TGF-β and IFN-α mediated inhibition of Fli1 levels [42]. Fli1 and ERG also play a critical role in suppressing inflammation in ECs, and their deficiency results in a robust production of inflammatory cytokines including IFN-β, IL-8, CXCL10, CX3CL1 and others [40]. Furthermore, injured endothelial cells express increased levels of adhesion molecules, including intercellular adhesion molecule-1 (ICAM-1), glycosylation-dependent cell adhesion molecule-1 (GLYCAM-1), vascular cell adhesion molecule-1 (VCAM-1), and E- and P-selectins (SELE and SELP) [35, 43], which facilitate immune cell recruitment (see below). In turn, infiltrating immune cells produce cytokines that further enhance inflammatory phenotype of ECs, including members of the IL-6 family such as IL-6 and Oncostatin M (OSM). These cytokines are potent inducers of other inflammatory cytokines in ECs through activation of the JAK/STAT3 signaling pathway (unpublished observations).

Therefore, in SSc, ECs are the primary site of inflammation that propagates the downstream manifestation of fibrosis. In other fibrotic diseases such as end-stage kidney disease and liver cirrhosis, initial tissue injuries may occur in the organ parenchyma, which provides the initial wave of inflammatory cytokines, but dysfunctional vasculature constitutes a major component in disease manifestation. In kidney disease, insults to the kidney trigger the endothelium to switch from a quiescent to an activated state, which leads to a cascade of pathways that contribute to fibrosis [44, 45]. These cellular changes can be reproduced in an experimental system, in which human coronary arterial endothelial cells were propagated and treated with recombinant human IL-1β [46]. Microarray transcriptome analysis of the treated endothelial cells show upregulation of genes previously observed in other fibrotic systems, including COX-2, TNF-α, IL-6, IL-1β, MMP-1, GM-CSF, NF-kB, c-Rel, VCAM-1, ICAM-1, SELE, etc. Among these, VCAM-1, ICAM-1 and SELE are leukocyte adhesion molecules that promote leukocyte capture and extravasation. These molecules promote the adhesion of inflammatory cells such as monocytes, neutrophils, lymphocytes, and macrophages that contribute to additional cytokines, growth factors and matrix metalloproteinases (MMPs). Expression of these adhesion molecules and cytokines suggests that EC activation plays an integral role in homing of macrophages and other inflammatory cells, contributing to tissue inflammation. Activated ECs can also cause detachment or activation of perivascular cells such as pericytes that can transition to myofibroblasts, further promoting fibrosis and destabilization of vasculature [47].

### The role of dysfunctional ECs in fibrosis

As described above, ECs can orchestrate tissue response to injury via secretion of proinflammatory and profibrotic cytokines [48, 49]. In addition, recent studies indicate that ECs may also contribute to perivascular ECM remodeling by transitioning to mesenchymal cells through the process of endothelial-to-mesenchymal transition (EndoMT). EndoMT was first observed in the developing embryo, in which ECs in the endocardium undergo EndoMT to invade the cardiac jelly and generate the cardiac cushions [50–52]. Similar to epithelial-to-mesenchymal transition (EMT), EndoMT can be triggered in adult tissues under certain pathological conditions such as inflammation, giving rise to myofibroblasts and contributing to fibrosis [53]. The presence of EndoMT was reported in several animal models of inducible fibrosis [53] as well as the skin and lungs of scleroderma patients [54]. However, the extent of contribution of EndoMT to the pathogenesis of human fibroproliferative diseases remains controversial. TGF-β is considered the primary inducer of EndoMT, but the involvement of other agonists has also been reported. For example, during cardiac infarction, activation of the Wnt/β-catenin pathway was shown to induce transition of ECs to the α-SMA-positive cells [55]. The Wnt/β-catenin pathway was also implicated in inducing EndoMT by the complement fragments C3a/C5a in a model of diabetic kidney disease [56]. A number of studies drew attention to the inflammatory cytokines that may be inducers of EndoMT. For example, a short-term exposure of valve endothelial cells to IL-1β or TNF-α induced transient EndoMT, while a longer exposure induced permanent transformation to myofibroblasts [57]. Similarly, IL-1β greatly potentiated TGF-β-induced EndoMT in HUVECs [58]. In cardiac valve endothelial cells, TNF-α or IL-6-induced EndoMT was mediated through activation of the Akt and NFκB pathways [59]. TGF-β promotes EndoMT through a complex mechanism that involves both Smad-dependent and Smad-independent pathways [60]. Notably, Jimenez and colleagues have shown that c-Abl and PKCδ mediate EndoMT in murine pulmonary ECs [61]. This profibrotic signaling pathway is known to downregulate transcription factor Fli1 in dermal fibroblasts and ECs [62, 63]. Recent studies have demonstrated that a combined knockdown of ERG and Fli1 in HUVEC induced EndoMT [40, 64]. Interestingly, the authors of the latter study also found downregulation of endothelial ERG and Fli1 within tumor stroma, likely caused by tumor secreted factors.

Thus, in some fibrotic diseases such SSc, ECs are the primary injured site and can generate subsequent inflammatory responses. In other cases, inflammatory cytokines can act on ECs, and ECs in turn respond by secreting additional cytokines and express adhesion molecules that further attract immune cells. In addition, they can also transform into mesenchymal cells and modify ECM, ultimately resulting in fibrosis.

**Fig. 1:**
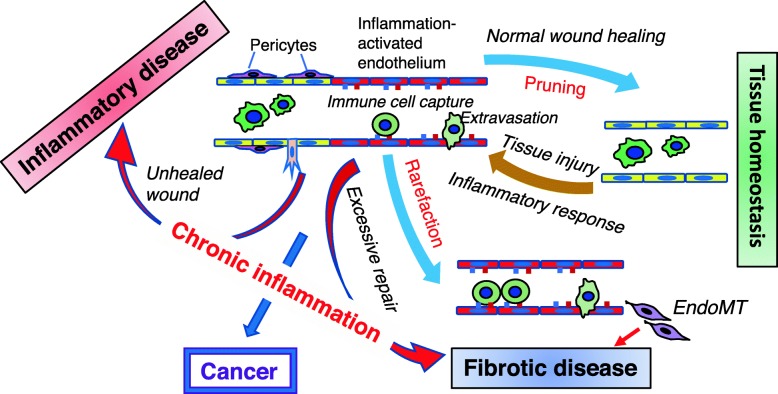
Proposed relationship between activated vasculatures found in injured tissue, inflammatory disease, fibrotic disease, and cancer. Injured tissue activates endothelium, which undergoes inflammatory responses and structural changes, including proliferation, loss of pericyte coverage, decreased junctional components and increased immune cell adhesion, that facilitate neoangiogenesis (shown as a sprout) and extravasation. In normal wound healing, inflammation-induced neoangiogenesis eventually ceases and the vessel undergoes pruning, and the tissue returns to homeostasis. In chronic inflammatory conditions, either the injury is never resolved, resulting in continued angiogenesis and chronic inflammatory disease, or the endothelium undergoes rarefaction and EndoMT, resulting in excessive repair and fibrotic disease. Cancer vasculature, while also considered a result of chronic inflammation, exhibits characteristics found in both inflammatory disease and fibrotic disease

The contrasting inflammatory responses in inflammatory disease and fibrotic disease mediated by dysfunctional ECs are summarized in Fig. 1.

### Vascular activation and tumorigenesis

Aggressive growth of neoplastic cell population can lead to hypoxic foci and overexpression of pro-angiogenic factors, which result in the development of disorganized blood vessel networks that are functionally and structurally different from normal vasculature. They are tortuous, hyperpermeable, and proliferative [65–67]. The essential roles of neoangiogenesis in tumor progression has prompted the development of antiangiogenic target therapies that remain the most important first-line treatment of many cancers such as renal cell carcinoma. However, these therapies have shown mixed results, with initial reduction in tumor burden, but often without improvement of long-term patient survival and in worst scenario resulting in increased metastasis [68, 69].

Some of these unsatisfactory outcomes have been attributed to a paradoxical success of the treatment: reduced vasculature in the tumor proper also hindered delivery of drugs to the lesion and might decrease infiltration of anti-tumor immune cells. In other cases, anti-angiogenic therapy can induce a drug-resistance response such as reduced expression of VEGF receptor (see below). A better understanding of tumor-associated vasculature is therefore needed. Recently, a more complex role of tumor vasculature has been proposed. As the host immunity can be both anti- and pro-tumorigenic, vasculature as a major player in tissue inflammation, can potentially also participate in the dichotomy of modulating tumorigenicity. Although the causal link between inflammation and cancer has been well accepted [70, 71], definitive data are still lacking concerning whether inflammation-activated vasculature plays any roles in cancer progression. Here we review the current knowledge of inflammation-activated vasculature and discuss how it may influence tumor progression.

The association of tumor with inflammation has been proposed in mid-nineteenth century by Rudolf Virchow, and the concept was reintroduced by H. F. Dvorak more than a century later [72]. Since then, accumulated evidence has indeed implicated inflammation in the development of various cancers [73, 74]. In particular, the development of renal cell carcinoma (RCC) has recently been linked to tissue inflammation [75–78]. There is emerging evidence that considers chronic kidney disease (CKD) and renal carcinoma as interrelated, with 26–44% of RCC cases bearing concomitant moderate or higher CKD at the time of diagnosis. In one large scale study involving over one million individuals, worsening kidney functions in CKD patients correlates significantly with increased risk of developing RCC [79]. In addition, patients suffering from renal cancer are more predisposed to CKD than the general population [80]. Therefore anti-inflammatory therapy may also work in RCC.

### Signaling pathway

As discussed above, during inflammation and wound healing, initial waves of proinflammatory cytokines stimulate endothelial cells to upregulate adhesion molecules and cytokines that together attract additional immune cells and mediate their capture and extravasation from blood vessels to the damaged tissue. TNF-α is a major mediator of inflammation in the tumor microenvironment during early tumorigenesis [81], controlling a cascade of cytokines, chemokines, adhesion molecules, and pro-angiogenic activities [81, 82]. The role of TNF-α signaling in tumor progression was demonstrated by comparing the growth of B16-F1 murine melanoma xenograft in wild-type (WT) mice and mice with germline deletions of both TNF-α receptors (TNFR 1 and 2) [82]. The growth of B16-F1 tumor was more than two-fold slower in TNFR knockout than in wild-type. Gene profiling performed on the endothelial cells isolated from the B16-F1 tumors showed that a majority of enriched EC genes from the wild-type stroma was involved in immune response. Pathway analysis showed that they were regulated by TNF-α signaling mediated by NF-κB and interferons.

The best-characterized actions of malignant cell-derived TNF-α are on vascular endothelial cells. Franses et al. has shown that TNF-α, together with angiogenic factors VEGF and FGF2, can induce a dysfunctional endothelial cell phenotype including increased proliferation, monolayer permeability, and monocyte adhesion [83]. These endothelial cells also exhibited a significant increase in NF-κB-p65 and pSTAT3 nuclear localization, suggesting a sustained pro-inflammatory state. The inflammatory phenotype was also confirmed in the global gene expression pattern, including up-regulation of GM-CSF, IL-8, IL-6 and SELE, while the expression of quiescence-promoting, anti-inflammatory genes such as eNOS, VE-cadherin, and Ang1 was decreased. Concurrently, the expression of leukocyte adhesion molecules VCAM-1 and ICAM-1 was also increased.

Besides TNF-α, other cytokines such as IL-1β, IL-6 and IL-17 have also been shown to induce vascular activation [84]. Importantly, IL-1β, IL-6 and TNF-α have been consistently found to over-express in clear-cell RCC (ccRCC) clinical samples and animal models [85–89]. Therefore, cancer cells in general and ccRCC cells specifically can produce cytokines that induce vascular activation.

TNF-α signaling is mediated by the activation of NF-κB and c-Jun N-terminal kinase (JNK). TNF ligand binding to the receptor causes dissociation of the inhibitory protein SODD from the intracellular death domain, followed by the binding of the adaptor protein TRADD, which serves as a platform for recruiting another protein TRAF2. TRAF2 then binds to kinases such as IKK, ASK, and MEKK1, leading to activation of NF-κB, JNK, and p38 MAP kinase. Activated NF-κB and JNK are the two major inducers of inflammatory response genes.

Canonical IL-6 signaling involves ligand binding to a type I cytokine receptor complex consisting of IL-6Rα chain and the signal-transducing component gp130 [90]. Gp130 is also the common signal transducer for several IL-6 family of cytokines including leukemia inhibitory factor (LIF), ciliary neurotropic factor, Oncostatin M, IL-11 and cardiotrophin-1. IL-6 binding triggers dimerization of gp130 and IL-6R, activating the receptor. Activated IL-6R-gp130 complex initiates a signal transduction cascade through activation of Janus kinases (JAKs) and transcription factor Signal Transducers and Activators of Transcription (STATs) [91]. Activated JAKs phosphorylate themselves and the receptor. The phosphorylated sites on the receptor and JAKs serve as docking sites for the SH2-containing STATs, such as STAT3, and for other SH2-containing proteins and adaptors that link the receptor to MAP kinase, PI3K/Akt, and other cellular pathways.

Gp130 is ubiquitously expressed in most tissues. In contrast, the expression of IL-6Rα is more restricted. For example, ECs do not express membrane-bound IL-6 receptor; instead, IL-6 signaling in ECs requires a mechanism called trans-signaling [92–94]. A soluble form of IL-6R (sIL-6R) comprising the extracellular portion of the receptor can bind IL-6 with a similar affinity as the membrane bound IL-6R. The complex of IL-6 and sIL-6R can then bind to gp130 on ECs and initiate signaling. One important supplier of sIL-6R is neutrophils during the initial phase of immune response [95]. Activated CD4 T cells is also a potential producer of sIL-6R [96]. It is not yet clear which cell types produce sIL-6R in the context of vascular activation in cancer.

Another inflammatory cytokine Oncostatin M (OSM) has been recognized as an important activator of ECs [97, 98]. OSM is a member of the IL-6–related family of cytokines that share receptor components [99]. It is suggested that endothelial cells are likely the prime target of OSM in vivo because endothelial cells express the highest levels of OSM receptors (10–20 fold higher) compared to all other cell types. The OSM receptor level is also much higher than that of TNF-α receptors on ECs [100, 101]. Analysis of The Cancer Genome Atlas (TCGA) data set shows that OSMR is expressed highly in many cancers, including renal, breast, lung, and liver cancers, and is associated with reduced survival rate (https://www.proteinatlas.org/ENSG00000145623-OSMR/pathology).

OSM can be produced by stimulated T-cells, monocytes, and rolling neutrophils [102, 103]. The effects of OSM on ECs suggest a pro-inflammatory role for OSM; for example, stimulation of the cultured endothelial cell HUVEC with human OSM results in prolonged upregulation of P-selectin [104], which facilitates leukocyte adhesion and rolling. OSM also promotes the production of IL-6 from these cells [102]. Interestingly, both SELE and SELP can promote metastasis of colon cancer cells [105].

### Markers of inflammation-activated vasculature

Activated ECs in the inflamed tissue express and secrete a spectrum of inflammatory cytokines and chemokines that further enhance the inflammatory microenvironment [82]. In one study, MCP-1, IL-6, GRO, IL-8, RANTES and GM-CSF were identified in the conditioned media of endothelial cells (HUVEC) induced by TNF-α and angiogenic factors [83]. Wang et al. also found that in a mouse glioblastoma model, ECs are the major source of IL-6 that induces macrophage M1-M2 polarization [106]. Importantly, these activated ECs also exhibit distinct cellular phenotypes that contribute to tissue inflammation, including an increase in the expression of adhesion molecules such as selectins, VCAM-1, and ICAM-1, as described above. These ECs also show decreased expression of VE-cadherin and CD31, which facilitate extravasation of the rolling leukocytes [83, 107, 108]. Importantly, these molecular and cellular features can presumably also promote cancer cell dissemination and metastasis.

In support of the notion that inflammation-activated or dysfunctional vasculature occurs in cancer progression, circulating levels IL-6 and TNF-α have been correlated with the risk of developing cancer, and with increased cancer deaths among older adults [109]. In addition, the activated EC markers SELE, ICAM-1 and VCAM-1 have been correlated with reduced ccRCC survival (Human Protein Atlas).

### Potential roles of dysfunctional vasculature in tumor progression

The inherent technical challenge of studying tumor microenvironment is the limited options of animal models. Traditional xenograft model using immune-compromised mice is inadequate for studying the impact of immune components on cancer progression. As such, most of the studies on tumor-related, inflammation-induced dysfunctional vasculature employed congenic mouse tumor models, or utilized manipulated endothelial cells together with human cancer cells in the same xenograft. In human, tumorigenesis in the context of relevant vascular diseases has been assessed.

In a study by Pitroda et al., malignant human colon cancer cells were imbedded with conditioned media from TNF-α and IFN-treated or untreated HUVECs, and xenografted in athymic mice. Activated conditioned HUVEC media promoted tumor growth 2.3-fold faster than control HUVECs [82].

The relationship between ECs and tumor is in fact complex. Intact or healthy vasculature has been shown to inhibit cancer cell proliferation, invasiveness, and response to inflammatory mediators in vitro, as well as tumor growth and metastasis in vivo. Such regulatory circuit is mediated by paracrine actions emanating from ECs, suggesting an active role of these healthy ECs [110]. Interestingly, these authors subsequently showed that in vitro cancer cell proliferation and invasion could also be inhibited by dysfunctional ECs (activated by TNF-α, VEGFA, and FGF2), to an extent even greater than by the quiescent ECs [83]. Furthermore, using a congenic Lewis lung carcinoma (LLC) implantation-resection-metastasis model, it was demonstrated that quiescent and dysfunctional ECs imbedded in the matrix could both suppress the growth of neighboring imbedded primary tumor; whereas only dysfunctional ECs could stimulate spontaneous metastasis [83].

The above findings raised the question of whether the known disease of systemic vascular inflammation, vasculitis [111, 112], is associated with higher risk of malignancy. The complicating factor in such studies is that the potential link between vasculitis and cancer may be the result of the underlying autoimmune disorder and the therapies (often cytotoxic) against such abnormalities, or may be simply because of a more intensive examination regimen applied to these patients [113]. However, accumulated evidence does point to such a comorbidity association. In a 10-year (1992–2002) French retrospective study, 60 (age 22–89) of 557 vasculitis patients (> 10%) were diagnosed concurrently with malignancy (excluding malignancy secondary to vasculitis therapy), as compared with the cancer incidence of French general population in 2000 (0.5–0.6%) [114]. Among these 60 patients, 22 (36.7%) developed vasculitis and malignancy within 1 year.

Conversely, vasculitis as a manifestation of underlying malignancy has also been demonstrated. The most common vasculitic manifestation of malignancy is cutaneous vasculitis such as leukocytoclastic vasculitis. In a single-institution review of 2800 vasculitis patients seen over an 18.5-year period, 12 patients (0.4%) were diagnosed with vasculitis and cancer within 1 year [115]. The most common cancer-associated vasculitis was leukocytoclastic vasculitis (7 of 12 cases) that manifested systemically in the skin. Importantly, 8 of 10 cases with > 2 months’ follow-up showed concordance of treatment response for both cancer and vasculitis, including a few cases of normally steroid-responsive leukocytoclastic vasculitis that did not respond to prednisone until treatment was initiated for the underlying cancer. Such clinical concordance supports the notion that the two diseases are linked. Clinical concordance has also been reported in another study [116], in which 15 patients with vasculitis and concurrent solid tumors (within 1 year) were followed. In 13 of these 15 cases the vasculitis was improved upon treatment of cancer.

Not surprisingly, hematologic cancers such as lymphomas and leukemias are the most frequent malignancy associated with leukocytoclastic vasculitis. However, organ-specific association of, for example, renal vasculitis and renal cell carcinoma, has also been observed in various case reports [117, 118], as well as incidence of leukocytoclastic vasculitis that is resolved upon curative treatment of RCC [119].

Therefore vasculitis appears to be linked with malignancy, although such correlation needs additional clinical evidence and mechanistic explanation.

### EndoMT - does it play a role in tumorigenesis?

EndoMT has been demonstrated in wound healing [120], atherosclerosis [121], pulmonary arterial hypertension (PAH) [122], cardiac and renal fibrosis [53, 120, 123], scleroderma [124], and cancer progression [125]. More recently, Xiao et al. showed that a subpopulation of tumor endothelial cells from a spontaneous mouse mammary tumor could undergo EndoMT after treatment with TGF-β, while normal mammary ECs could not, suggesting tumor-associated ECs may be inherently sensitive to mesenchymal transition induction [126]. In a mouse glioblastoma model, the tumor-associated ECs can be induced to undergo EndoMT by human glioblastoma cell-conditioned media or PDGF-AB, which is accompanied by reduced VEGFR2 expression [127]. The authors argued that these transformed ECs might account for the resistance to anti-VEGF therapy.

Nonetheless, the pathological role of EMT and EndoMT remains controversial. One main reason has been the difficulty in demonstrating the event in vivo [123, 124]. Great efforts have been made in animal models using genetic manipulations and lineage tracing. However, the results have been inconsistent. For example, concerning the source of myofibroblasts in renal fibrosis, tubule epithelial cells, pericytes, endothelial cells, resident fibroblasts, and bone marrow-derived cells have been identified in different studies as the source, either exclusively or with varying percent contributions [123]. Marker selection and staining methods likely contribute to the inconsistency. For example, Tie-2-driven lineage may include monocytes, not exclusively endothelial cells, while PDGFR-driven lineage may include endothelial cells, not exclusively pericytes. As for EndoMT, one important criterion is the loss of endothelial cell markers such as CD31 and VE-cadherin. But without such markers, it would be difficult to confirm the dual characteristics of mesenchymal and endothelial cells outside of the endothelium. This is particularly problematic in human clinical samples, where lineage tracing is not possible.

As such, the recently noted phenomenon of partial EndoMT represents an alternative that is more readily verifiable [128, 129]. That is, during angiogenic and/or inflammatory responses, ECs retain intercellular junctional molecules such as CD31 and VE-cadherin, while expressing many of the mesenchymal makers such as vimentin or α-smooth muscle actin. Such condition can indeed be observed at the tips of sprouting capillaries, in which tip cells remain attached to the endothelium while exhibiting capability of motility and matrix degradation. It would be interesting to assess whether partial EndoMT is solely the result of neoangiogenesis or may be partly induced by inflammatory signals.

### Tumor vasculature: Neoangiogenesis vs. inflammation-induced vascular activation

It has been well-accepted that dysfunctional vasculature is an important component in tissue fibrosis. If one also accepts the notion that inflammation-induced vascular activation is part of tumorigenesis, it would be important to reconcile the apparent vascular phenotypes in fibrosis and tumor. In fibrosis, the vasculature is characterized, at least at the diseased state, by rarefaction, meaning loss of vasculature; whereas in tumor, neo-angiogenesis is the observed phenotype. Indeed, inflammation-activated vasculature has been shown to be non-proliferative. How, then, can tumor-associated vasculature be both proliferative and a response to inflammation? There are a few possibilities:

First, neo-angiogenic and inflamed ECs may coexist in different areas (e.g., hypoxic vs. normoxic) of the tumor microenvironment. This can account for the fact that inflamed vasculature is not proliferative.

Second, the two types of tumor vasculature may appear sequentially during cancer progression; that is, it is possible that tumor-induced angiogenesis occurs early and inflammation is initiated later for pruning the tumor-associated vessels and facilitating metastasis. The report that inflamed ECs inhibit primary tumor growth but promote metastasis supports this notion [110]. However, the reverse temporal sequence is also possible since in wound healing, inflammation usually precedes proliferation (anti-infection followed by wound repair) [130]. Interestingly, in the peritoneal inflammation model, IL-6 together with sIL-6R treatment of peritoneal mesothelial cells could induce VEGF expression and promote angiogenesis [131].

Third, tumor-induced vessels are inherently different from the inflammation-activated vessels; the former could be both proliferative and inflammatory. In the report that showed activated HUVECs could stimulate malignancy, the HUVECs actually required stimulation of both TNF-α and angiogenic factors such as VEGF and FGF2 [83]. In fact, neoangiogenic ECs and inflamed ECs share at least the same characteristic of reduced expression of cell-cell junction components. This can also explain the partial EndoMT phenotype, and may unify the requirement for both neoangiogenesis and inflammation-activated vasculature in cancer progression.

## Conclusion

**Fig. 2:**
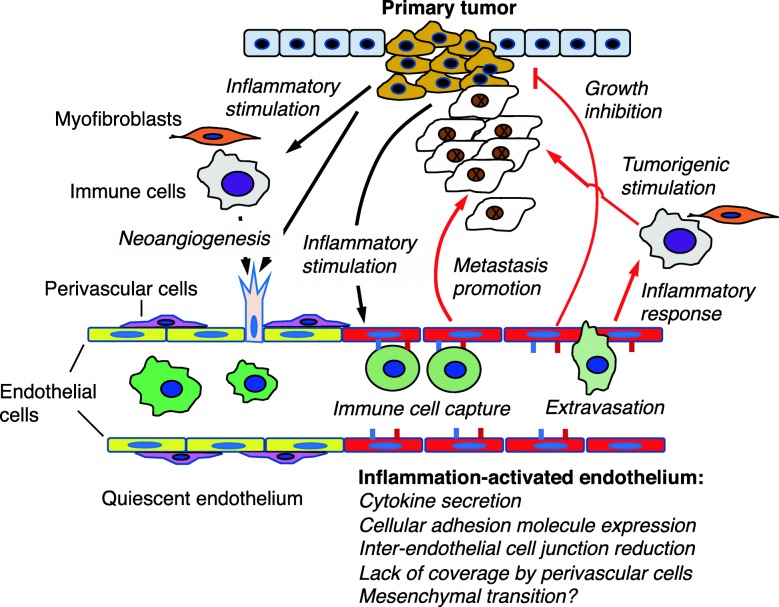
Summary of interaction between tumor and tumor-associated vasculature. Tumor cells can induce inflammatory response in myofibroblasts, tissue resident immune cells, and endothelial cells (black arrows). Both tumor cells and activated stromal cells can induce neoangiogenic response. Inflammation-activated endothelial cells in turn can respond by secreting cytokines to activate other stromal cells, and express adhesion molecules (red and blue short bars) that allow capture of circulating immune cells (green), and facilitate their extravasation. Activated endothelial cells, which lack coverage of perivascular cells, can also inhibit primary tumor growth while promoting metastasis

It has been recognized that the endothelium is an integral part of tissue inflammation and fibrosis. Most often, the endothelial cells are activated by the cytokines secreted by the injured tissues and immune cells, and they in turn respond by expressing cytokines and adhesion molecules that exacerbate the inflammatory response. In the cases such as SSc and vasculitis, the endothelium is likely the primary injured tissues, and it responds by secreting inflammatory cytokines to initiate the inflammatory response. Regardless of the initial injury, in both scenarios ECs play a critical role in the inflammatory process. In fibrosis, it has been proposed that EndoMT is a key mechanism that provides myofibroblasts; although its pathophysiological role in human diseases requires further verification. The most potent cytokines that engage ECs appear to be TNF-α, IL-6, IL-1β, and OSM, while activated ECs also produce TNF-α and IL-6, thus forming a self-perpetuating loop. Interestingly, the similar intercellular interactions likely also occur in the tumor microenvironment. The interaction between tumor cells and tumor-associated endothelial cells is summarized in Fig. 2. The question arises as to how the self-perpetuating mechanism is broken in healthy inflammatory response such as wound healing. One possibility may be the balance between neoangiogenesis and vessel maturation (pruning). Neoangiogenesis is needed at the beginning of wounding response, but vessel maturation and pruning are required at the end of wound repair process. On the other hand, fibrosis (the result of chronic inflammation) is often accompanied by vessel rarefaction while in cancer, neoangiogenesis is essential. In such scenario, cancer-associated vasculature may be considered a hybrid of chronic inflammatory mediator that shares characteristics with vasculatures in inflammatory disease and fibrotic disease (Fig. 1). Future studies that elucidate these seemingly contradictory roles of dysfunctional ECs in different inflammatory conditions, including cancer, will be critical for designing new therapies against these, at present incurable maladies.

## Data Availability

Not applicable.

## Abbreviations

AECA: Anti-endothelial autoantibody
CKD: Chronic kidney disease
EC: Endothelial cell
ECM: Extra-cellular matrix
EMT: Epithelial-to-mesenchymal transition
EndoMT: Endothelial-to-mesenchymal transition
FGF: Fibroblast growth factor
GLYCAM: Glycosylation-dependent cellular adhesion molecule
GM-CSF: Granulocyte-macrophage colony-stimulating factor
GRO: Growth-regulated
HUVEC: Human umbilical vein endothelial cell
IBD: Inflammatory bowel disease
ICAM: Intercellular adhesion molecule
IL: Interleukin
JAK: Janus kinase
JNK: c-JUN N-terminal kinase
MCP-1: Monocyte chemoattractant protein-1
MMP: Matrix metalloproteinase
OSM: Oncostatin M
RA: Rheumatoid arthritis
RANTES: Regulated on activation, normal T cell expressed and secreted
RCC: Renal cell carcinoma
SSc: Systemic sclerosis
STAT: Signal transducer and activator of transcription
TGF: Transforming growth factor
TNF: Tumor necrosis factor
VCAM: Vascular cell adhesion molecule
VEGF: Vascular endothelial growth factor
